# Sortilin Expression Levels and Peripheral Immunity: A Potential Biomarker for Segregation between Parkinson’s Disease Patients and Healthy Controls

**DOI:** 10.3390/ijms25031791

**Published:** 2024-02-01

**Authors:** Maria Georgoula, Panagiotis Ntavaroukas, Anastasia Androutsopoulou, Georgia Xiromerisiou, Fani Kalala, Matthaios Speletas, Eftihia Asprodini, Anna Vasilaki, Stamatia Papoutsopoulou

**Affiliations:** 1 Department of Biochemistry & Biotechnology, University of Thessaly, 41500 Larissa, Greece; marigeorgoula@uth.gr (M.G.); pntavaroukas@uth.gr (P.N.); anastasiandr2000@gmail.com (A.A.); 2 Faculty of Medicine, University of Thessaly, 41500 Larissa, Greece; georgiaxiromerisiou@gmail.com; 3 Laboratory of of Immunology & Histocompatibility, Faculty of Medicine, University of Thessaly, 41500 Larissa, Greece; fkalala@uth.gr (F.K.); mspeletas@uth.gr (M.S.); 4 Laboratory of Clinical Pharmacology, Faculty of Medicine, University of Thessaly, 41500 Larissa, Greece; easpro@uth.gr; 5 Laboratory of Pharmacology, Faculty of Medicine, University of Thessaly, 41500 Larissa, Greece; a.vasilaki@uth.gr

**Keywords:** sortilin, Parkinson’s disease, peripheral immunity, monocytes

## Abstract

Parkinson’s disease (PD) is characterized by substantial phenotypic heterogeneity that limits the disease prognosis and patient’s counseling, and complicates the design of further clinical trials. There is an unmet need for the development and validation of biomarkers for the prediction of the disease course. In this study, we utilized flow cytometry and in vitro approaches on peripheral blood cells and isolated peripheral blood mononuclear cell (PBMC)-derived macrophages to characterize specific innate immune populations in PD patients versus healthy donors. We found a significantly lower percentage of B lymphocytes and monocyte populations in PD patients. Monocytes in PD patients were characterized by a higher CD40 expression and on-surface expression of the type I membrane glycoprotein sortilin, which showed a trend of negative correlation with the age of the patients. These results were further investigated in vitro on PBMC-derived macrophages, which, in PD patients, showed higher sortilin expression levels compared to cells from healthy donors. The treatment of PD-derived macrophages with oxLDL led to higher foam cell formation compared to healthy donors. In conclusion, our results support the hypothesis that surface sortilin expression levels on human peripheral monocytes may potentially be utilized as a marker of Parkinson’s disease and may segregate the sporadic versus the genetically induced forms of the disease.

## 1. Introduction

Parkinson’s disease (PD) is a complex neurodegenerative disorder that predominantly affects the elderly population and is characterized by a range of motor and non-motor symptoms [1,2,3]. The classical pathological hallmarks of PD include the loss of dopaminergic neurons in substantia nigra and the presence of Lewy bodies, which are protein aggregates mainly composed of alpha-synuclein (α-syn) [4]. While the etiology of PD remains elusive, it is generally accepted that a combination of both genetic and environmental factors contributes to its onset and progression [5,6,7]. Traditionally, research in PD has been neuron-centric, focusing on the mechanisms leading to neuronal death and strategies to protect the vulnerable neuronal populations. However, a growing body of evidence suggests that the immune system plays a critical role in the pathogenesis of PD [8]. This concept is further supported by studies from both our and other groups that demonstrate increased levels of proinflammatory cytokines, such as tumor necrosis factor alpha (TNF), interleukin-6 (IL-6) or interleukin-1 (IL-1) in the serum of PD patients compared to healthy donors [9,10,11,12,13,14]. The main producers of proinflammatory cytokines in the periphery and in the brain are usually activated innate immune cells, such as monocytes, macrophages and microglia [14].

Several studies on PD patients have revealed that genetic predisposition impacts PD pathogenesis, especially in familial and early-onset cases. Genome-wide association studies (GWAS) have identified several genes as risk factors, including *SNCA* (encoding α-syn), *PRKN* (encoding parkin), *PINK1* (encoding PTEN-induced kinase 1) and *LRKK2* (encoding Leucine rich repeat kinase 2) [15,16,17]. Some of these proteins can modulate immune cell function, e.g., parkin regulates microglial NLRP3 inflammasome activation through polyubiquitination and thus alleviates neurodegeneration in PD [18]. In addition, α-syn has been shown to modulate immune cell function and its accumulation activates microglia and promotes neuroinflammation in PD [19,20]. A whole exome sequencing and linkage analysis study has recently identified, in a Greek family, a novel mutation in *SORL1* (or *SORLA)* gene that encodes sortilin-related receptor 1 [21]. SORLA is member of the vacuolar protein sorting-10 (Vps10) domain-containing receptor family that contains five members: SORLA, sortilin, SorCS1, SorCS2, and SorCS3 [22]. These proteins have been implicated in the pathophysiology of neurodegenerative and other diseases [23]. Sortilin is encoded by the *SORT1* gene, and a genetic variant of this gene was shown to be associated with a reduced risk of Alzheimer’s disease [24], although an earlier study did not show an association of *SORT1* gene polymorphism with sporadic Alzheimer’s disease in a specific population [25]. Bioinformatics and sequencing approaches showed a correlation of *SORT1* gene expression with PD [26] and essential tremors [27]. To date, only a few studies have shown that sortilin and SORLA may have unique functions in monocytes and macrophages, whereas SorCS1, SorCS2 and SorCS3 are expressed and function only in the brain. Sortilin and SORLA have been linked to immune system function and inflammation [28]. SORLA can modulate the migration of monocytes in vitro in stimulated THP1 cells with conjugated linoleic acids [29]. Studies on SORT1 knockout mouse [30] revealed that sortilin facilitates exocytic trafficking of interferon-gamma (IFN-γ) and Granzyme A by T lymphocytes in murine experimental models, as well as interferon-alpha (IFN-α) in CpG-A-stimulated plasmacytoid dendritic cells (pDCs) [28,30]. This raises the intriguing possibility that these proteins may serve as a link between neurodegeneration and immune activation in PD. Understanding the regulation of their expression in immune cells may provide valuable insights into disease mechanisms and offer novel avenues for therapeutic intervention. 

In this study, we have examined the lymphocytic and monocytic populations in peripheral blood from healthy donors and PD patients with sporadic disease or specific mutations. To the best of our knowledge, this is the first study that reveals a substantial increase in sortilin on monocytes from PD patients. This novel observation has been further tested on isolated peripheral blood mononuclear cell (PBMC)-derived macrophages that have been cultured in vitro and analyzed for activation marker expression and foam cell formation. Given the emerging evidence pointing to the involvement of the immune system in PD pathogenesis, the understanding of the expression and function of sortilin in innate immune cells may provide valuable insights into the disease mechanisms and pave the way towards novel therapeutic approaches.

## 2. Results

### 2.1. Patient Demographics of the Main Cohort Study

A novel cohort of patients was recruited that consisted of 11 healthy volunteers (7 males and 4 females) and 10 PD patients (4 males and 6 females) at the outpatient clinic at General Hospital of Larisa, Thessaly, Greece (Table 1). The PD patients were represented by five sporadic cases (PD_Sporadic) and five patients carrying known mutations on *SNCA* (one patient with A53T and one patient with A30P) and *SORLA* (three patients with G379W) (PD_Genetic). The three PD patients carrying the G379W mutation were recently identified and characterized and belong to a Greek family, showing that *SOLRA* mutations result in neurodegenerative phenotypes other than Alzheimer’s disease [21]. 

### 2.2. Altered Immune Cell Populations in Peripheral Blood of PD Patients

**Figure 1:**
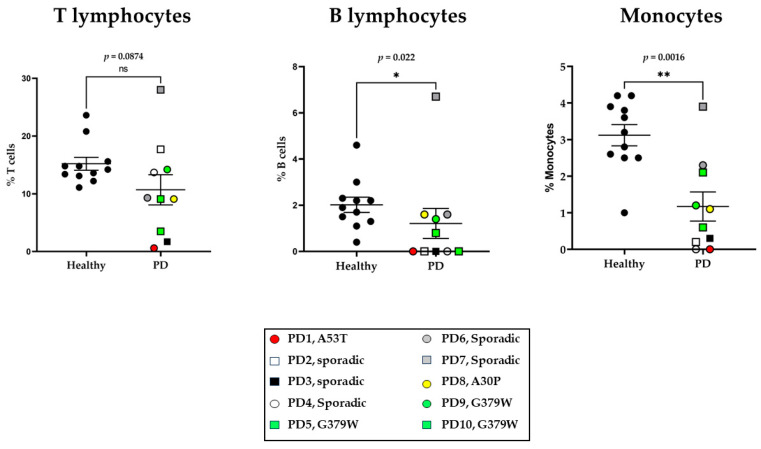
Flow cytometry analysis of peripheral immune cells. Percentage of main lymphocyte populations, CD3^+^ T cells and CD19^+^ B cells, a CD14^+^ monocytes in peripheral blood of healthy donors (N = 11) and in the PD patient groups (N = 10). Mann–Whitney non-parametric analysis was performed (* *p* < 0.05, ** *p* < 0.01). ns = non-significant.

One of the main targets of our study was to examine possible alterations in the numbers of cells within the main immune cell populations in the blood of PD patients compared to healthy donors. For this purpose, we performed flow cytometry on freshly isolated peripheral blood (Figure 1 and Supplementary Table S1). The cell numbers are expressed as a percentage of the total ungated cells, and the relevant strategy is presented in Supplementary Figure S1. There was no significant alteration in the numbers of CD3^+^ lymphocytes within the groups. Instead, B lymphocytes were low in PD patients, although their presence was clear in healthy donors (2.0 ± 0.3, as % of total blood cells). There was also a striking lower number of CD14^+^ monocyte population in PD patients. As presented in Figure 1, healthy donors show an average of 3.12 ± 0.29 CD14^+^ monocytes (as % of total blood cells), which declines to 1.17 ± 0.399 in PD patients, although we did not observe a difference between the sporadic and genetic PD cases. 

**Figure 2:**
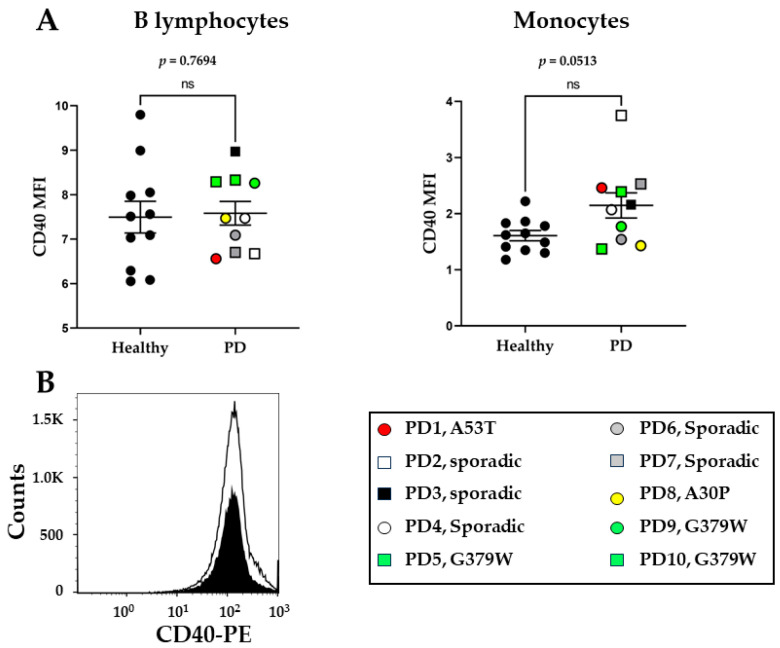
Flow cytometry analysis of CD40 surface levels on CD19^+^ B cells and CD14^+^ monocytes. Peripheral blood from healthy donors (N = 11) and PD patients (N = 10) were analyzed for CD40 expression. (**A**) MFI: mean fluorescence intensity of CD40 in B lymphocytes and monocytes. (**Β**) Representative histogram of CD40 expression in monocytes (healthy donors = black histogram, PD donors = white histogram). Mann–Whitney non-parametric test was performed. ns = non-significant.

We also examined the levels of CD40, the receptor that is expressed on antigen-presenting cells, such as B lymphocytes and monocytes, and interacts with the CD40 ligand (CD40L) expressed by activated T cells [31]. CD40 protein levels, expressed as mean fluorescence intensity (MFI), in B cells were similar between the two groups and there was no difference between the sporadic and genetic PD cases. In monocytes, there was a trend toward higher cell surface CD40 levels in PD patients, although this did not quite reach statistical significance, *p* = 0.0513 (Figure 2A). A representative histogram of CD40 levels on monocytes from healthy and PD patients is shown in Figure 2B.

### 2.3. Differential Sortilin Expression Levels on Monocytes from PD Patients and Correlation with Disease

**Figure 3:**
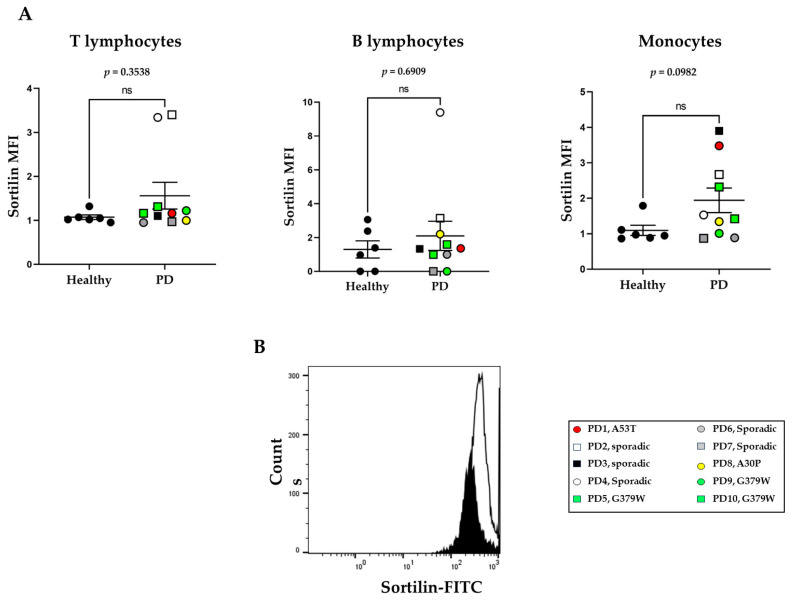
Sortilin protein expression on immune cells. Flow cytometry was applied for detection of sortilin on lymphocytes and monocytes in freshly isolated peripheral blood from healthy donors (N = 6) and in PD patients (N = 10). (**A**) Sortilin levels on CD3^+^ T cells, CD19^+^ B cells, and CD14^+^ monocytes in peripheral blood populations expressed as mean fluorescence intensity (MFI). (**B**) Representative sortilin expression histograms in monocytes (healthy = black histogram, PD = white histogram). Mann–Whitney non-parametric test was performed.

**Figure 4:**
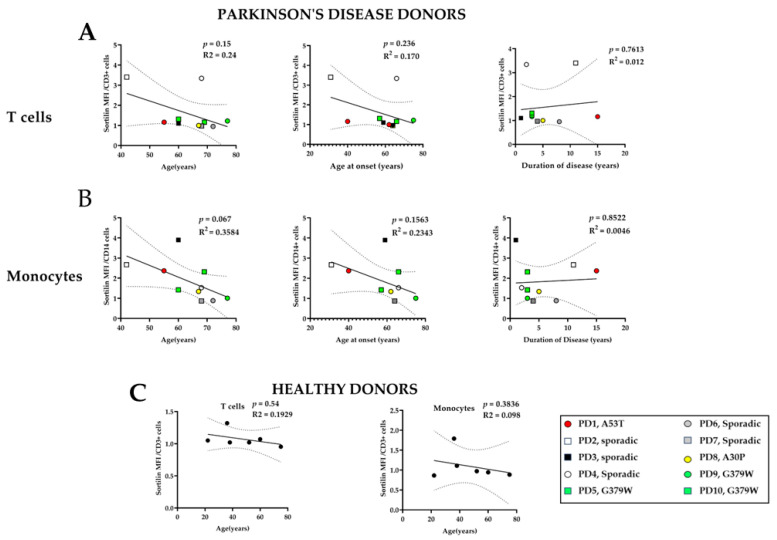
Linear regression analysis of sortilin levels in relation to age-related metrics. Sortilin levels, derived from flow cytometry data on CD3^+^ T cells (**A**) and CD14^+^ monocytes (**B**) from all PD patients, were analyzed in relation to patient age, age at disease onset and duration of disease. (**C**) Sortilin levels, derived from flow cytometry data on CD3^+^ T cells and CD14^+^ monocytes from healthy donors, were analyzed in relation to patient age. MFI: mean fluorescence intensity. ns = non-significant.

Peripheral blood monocytes were further analyzed for sortilin levels via flow cytometry (Figure 3A and Supplementary Figure S2). Sortilin levels on the cell surface of healthy monocytes were low (MFI: 1.094 ± 0.143). In monocytes, there was a trend toward higher cell surface sortilin levels in PD patients, although this did not quite reach statistical significance, *p* = 0.0982 (Figure 3A). A representative histogram of sortilin levels on macrophages from healthy and PD patients is shown in Figure 3B. We performed linear regression analysis as a simplified model to understand how sortilin levels expressed on the cell surface of peripheral immune cells might change in relation to the age of patient, age at onset, and the duration of the disease (Figure 4A,B). Sortilin levels in monocytes appear lower with increasing age of patients without reaching significance (*p* = 0.067), as shown in Figure 4A,B. Instead, in the healthy donors, we cannot observe any correlation of sortilin expression levels with age (Figure 4C). Sortilin expression appears to show a negative correlation with the age of onset in monocytes (*p* = 0.1363) and but no correlation with disease duration.

### 2.4. In Vitro Functional Studies Differentiate PBMC-Derived Macrophages between Healthy Donors and PD Patients

**Figure 5:**
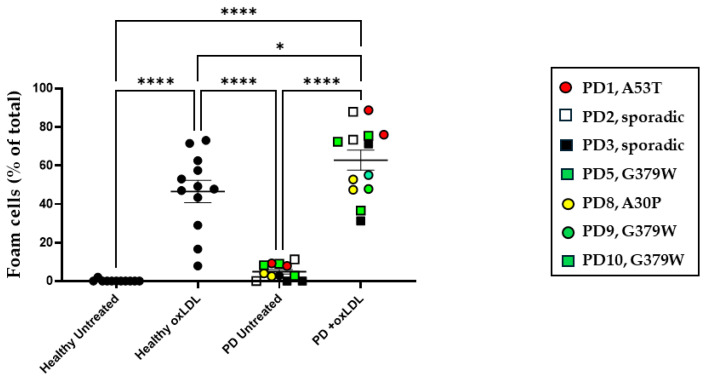
Foam cell formation in in vitro cultured macrophages. PBMC-derived macrophages from healthy donors (N = 7, n = 12) and PD patients (N = 7, n = 13) were cultured and either left untreated or treated with 10 μg/mL oxLDL for 24 h. Cells were stained with Oil Red O and the percentage of positive foam cells was calculated. Kruskal–Wallis analysis was performed with post hoc Dunn’s tests for multiple comparisons, (* *p* < 0.05, **** *p* < 0.0001).

**Figure 6:**
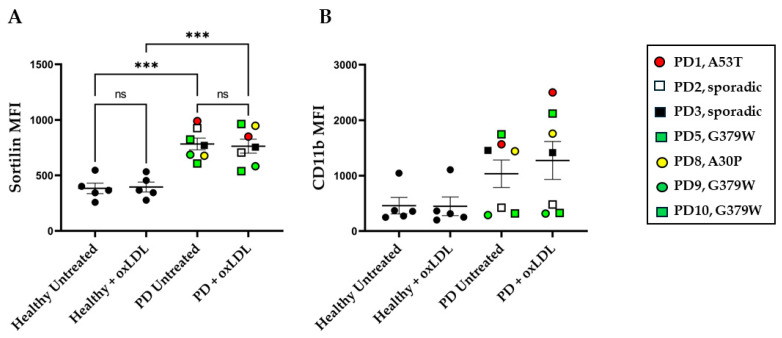
Flow cytometry analysis of in vitro cultured macrophages. PBMC-derived macrophages from healthy and PD donors (n = 5–7) were either left unstimulated or were treated with 10 μg/mL oxLDL for 24 h. Cells were then harvested and stained for surface sortilin expression (**A**) and CD11b expression (**B**). One-way ANOVA was used for statistical analysis. (*** *p* < 0.001). ns = non-significant.

The differential expression of sortilin on monocytes could potentially affect their biological functions. To investigate this hypothesis further, we isolated PBMC-derived monocytes from healthy donors and PD patients and differentiated them in vitro into macrophages. For this purpose, we used cells from highly sortilin-expressing PD patients, which were the PD_Genetic donors and two PD_Sporadic donors, as shown in Figure 5. The cells were consequently cultured in the presence or absence of oxidized-LDL for 24 h. Foam cell formation was determined via oil red staining and the percentage of positive cells was calculated based on microscopical examination (Figure 5 and Supplementary Figure S3). At steady state, the healthy cells show no cell formation, but the PBMC-derived cells from PD donors show approximately 4.8% foam cells, which is higher than that observed with healthy cells but not significantly different. In the presence of oxLDL for 24 h, higher foam cell formation was observed in cultures from PD patients (62.7%, *p* < 0.05) compared to healthy donors (43.6%). In parallel cultures, the samples were examined for cell surface expression of sortilin and CD11b via flow cytometry (Figure 6). The sortilin levels appeared to be higher on the cell membrane of cultured macrophages derived from PD donors compared to cells from healthy individuals (Figure 6A). We did not detect differences in the sortilin levels between PD_Sporadic and PD_Genetic in vitro cultured PBMC-derived macrophages; therefore, we present the data as one group. These data confirm our hypothesis that in PD patients, the sortilin levels are not only higher in blood monocytes, but they are also maintained at high levels in PBMC-derived macrophages during in vitro cultures. oxLDL, though, did not alter the expression levels of sortilin in either healthy or PD-derived cells. CD11b expression was similar in all cultures and was not affected by oxLDL treatment (Figure 6B).

## 3. Discussion

The role of the immune system in Parkinson’s disease is increasingly recognized as a critical factor in both disease onset and progression. While the primary focus of PD research has traditionally been on the central nervous system, emerging evidence suggests that peripheral immune cells also contribute significantly to the disease pathology. Studies on both human samples and experimental animal models have shown that peripheral immune cells, including T cells and monocytes, infiltrate the brain [32,33,34]. There are also observations of alterations in immune population numbers in the peripheral blood of PD patients. Yan et al. found that naive CD4^+^ and CD8^+^ T cells are significantly decreased in early-stage PD patients [35]. In that study, total CD19^+^ B cells were not affected, and the same was reported by others, as discussed by Jiang et al. [36]. In our study, we found reduced numbers of total CD19^+^ B cells in PD patients. This could be potentially explained by the treatment followed by these patients, as it has been shown that antiparkinsonian drugs like l-DOPA and apomorphine have an antiproliferative, cytostatic effect on B cells [37]. The same was shown in a study that assessed PD patients before and after the onset of levodopa treatment and showed a significant reduction in the numbers of CD19 ^+^ cells post the commencement of medication [38]. Regarding the monocyte population, a first in-depth analysis showed no difference between healthy controls and PD patients [39]. Instead, that study identified an enrichment in the CD14^+^CD16^−^ classical monocytes in the blood of PD patients [39]. In this study, we initially aimed to study the general CD14^+^ monocyte population and, surprisingly, we observed a significantly low number of blood monocytes in PD patients compared to healthy donors. We did not, however, see differences between the two PD groups (the PD_Sporadic group and the PD_Genetic group). The reasons behind this observation are unknown and further studies with more patients are required to identify the factors that lead to the disappearance and/or defective production or differentiation of monocytes in PD patients. The surviving monocytes, though, showed higher, although not statistically significant, levels of CD40, implying an activation status compared to the healthy donors that could be explained by higher activation levels of the transcription factor NF-κB, the major proinflammatory regulator of transcription in monocyte/macrophage populations [40]. 

One of the most interesting findings of the present study was the expression levels of sortilin. The sortilin expression levels on monocytes from PD patients were high, although not statistically significant, compared to those of healthy donors. Linear regression analysis revealed that the sortilin levels on monocytes showed an almost statistically significant negative correlation with the age of the PD donors, which was not observed in healthy individuals. These observations allowed us to speculate that sortilin might play a role in the immune system in Parkinson’s disease. Sortilin, or neurotensin receptor-3, is a type I transmembrane glycoprotein and a member of the VPS10P (Vacuolar Protein Sorting 10 Protein) family of receptors, which consists of five members with common features with the VPS10P domain that enables signal transduction, intracellular trafficking and sorting of proteins [22]. The most studied functions of sortilin that link this receptor to neurodegenerative diseases are the neurotrophic factor signaling, which contributes to neuronal viability and functionality and intracellular protein sorting [41,42,43]. Other studies, especially genome-wide association studies (GWAS), have linked sortilin expression and function and SorCS1 to glucose metabolism and the risk of type 2 diabetes development [44,45,46]. GWAS approaches suggest that the *SORT1* gene carries the strongest association with low-density lipoprotein cholesterol (LDL-C) of all loci, suggesting a role of sortilin in metabolic and cardiovascular diseases [43,47]. In contrast, much less is known about sortilin function in immune cells. Herda et al. showed that sortilin contributes to exocytic trafficking of interferon-γ and granzyme A in T cells, contributing to adaptive immune activation [30]. In macrophages, sortilin was shown to facilitate the secretion of interleukin-6 and interferon-γ in lipopolysaccharide-stimulated cells in vitro [48]. The same result was also shown in plasmacytoid dendritic cells by Yabe-Wada et al. [49]. Sortilin has been characterized as an APO E (apolipoprotein E) receptor that is responsible for the cellular uptake of APOE-containing lipoproteins in most cell types [50]. Moreover, experiments on *Sort1* knockout mice that lack sortilin revealed that sortilin in immune cells attenuated the inflammatory response and reduced atherosclerosis [48]. To further examine the role of sortilin in macrophages, we performed in vitro experiments using PBMC-derived macrophages from healthy donors and PD patients. Our results clearly showed that PBMC-derived macrophages from PD patients express higher levels of sortilin at steady state in unstimulated conditions compared to the ones derived from healthy donors. Those macrophages, though, responded to oxLDL treatment in a similar way to healthy donors and showed no further upregulation of sortilin. It is noteworthy to mention that this observation is different to that of a previous study on liver sinusoidal endothelial cells that showed that, upon oxLDL treatment, these downregulate sortilin expression [51]. Upon oxLDL treatment, the macrophages from PD donors that express higher levels of sortilin, compared to healthy donors, lead to the formation of a much higher number of foam cells. This is important as cholesterol-loaded cells, the so-called foam cells, are the hallmark of atherosclerosis [52]. oxLDL can alter the CD11b protein levels in innate immune cells, as was shown in monocyte cultures from total blood [53]. Conversely, in the present study, in which PBMC-derived macrophages were used, no downregulation of CD11b in oxLDL-treated macrophages was observed, perhaps due to different experimental conditions. The sortilin-mediated uptake of native LDL into macrophages may be an important mechanism of foam cell formation and contributor to atherosclerosis development [54,55]. 

The limitation of our study is the low number of patients that could inevitably impact the flow cytometry results from peripheral blood. On the other hand, this is a proof-of-concept study that shows that the expression of sortilin in immune cells and specifically in monocytes correlates with the age-related characteristics of PD patients. Moreover, peripheral monocytes and PBMC-derived macrophages from the same individuals show a similar sortilin expression profile, which correlates with foam cell formation upon oxLDL treatment in vitro. Future research could further investigate the molecular mechanisms that regulate the function of sortilin in innate immune cells in relation to PD. Moreover, scientists should aim to explore the potential therapeutic implications of modulating the immune function by targeting specific immune cell populations and offer new avenues for treatment. Given the complexity of the immune system and its intricate relationship with PD, multidisciplinary approaches that combine immunology, neurobiology, and bioinformatics are likely to be the most fruitful.

## 4. Materials and Methods

### 4.1. Study Participants

In this study, 11 healthy volunteers (7 males and 4 females) and 10 PD patients (4 males and 6 females) were enrolled at the outpatient clinic of General Hospital of Larisa, Thessaly, Greece. The diagnosis of PD was fulfilled in accordance with clinical diagnostic criteria of the UK Parkinson Disease Society Brain Bank. The PD patients consisted of 5 patients with sporadic disease (PD_Sporadic) and 5 patients carrying known mutations on *SNCA* (1 patient with A53T and 1 patient with A30P) and *SORLA* (3 patients with G379W) (PD_Genetic). Healthy controls, with an overall age and sex distribution like that of the patients, were recruited from the patients’ family (spouses) or from individuals who were willing to participate in this research. This study was approved by the local Ethics Committee 43/08.07.2022 and informed consent was obtained from all participants.

### 4.2. Blood Samples and Flow Cytometry Analysis

Peripheral venous blood (5 mL) was collected from the patients and healthy controls in EDTA tubes. All samples were kept at 4 °C and analyzed within 8 h. Then, 100 μL of blood was mixed with 1 mL VersaLyse Lysing Solution (Beckmann Coulter, Marseille, France) for red blood cell lysis. After a wash in PBS, the cells were incubated for 15 min at 4 °C with 10 μg FcR blocking antibody followed by incubation (30 min at 4 °C) with antibodies specific for T lymphocytes (1:400, anti-human CD3 PE), B lymphocytes (1:400, anti-human CD19 PC7) and monocytes (1:400, anti-human CD14 PC5), all purchased from Beckman. Sortilin was detected with anti-human sortilin unconjugated antibody (1:100, R&D Systems, Abingdon, UK) followed by mouse F(ab2) IgG (H + L) FITC (0.5 mg/mL, R&D Systems). Mouse IgG1 isotype control (0.5 mg/mL, R&D Systems) was used as negative control for this staining. Anti-human CD40 PE (1:100, Beckman Coulter, Marseille, France) was used to stain antigen-presenting cells in the periphery and anti-human CD11b (1:100, eBioscience, Hatfield, UK) was used for PBMC-derived macrophage staining. The cells were washed twice with PBS/1% FBS, resuspended in 0.5 mL PBS/1% FBS, and subsequently analyzed using the flow cytometer Cytomics FC 500 Beckman Coulter. Raw data analysis was performed using the CXP (Beckman Coulter, Marseille, France) and FlowJo™ v10.8 Software (BD Life Sciences, Franklin Lakes, NJ, USA).

### 4.3. Human Peripheral Blood Mononuclear Cell-Derived Macrophages—Isolation and In Vitro Differentiation

Peripheral venous blood (10 mL) was collected from patients and healthy controls on EDTA tubes. PBMCs were isolated as previously described [56] and they were either used directly or were frozen in aliquots. During the freezing process, PBMCs were aspirated, washed with sterile PBS, resuspended in 1 mL freezing medium (88%*v*/*v* FCS (GIBCO) plus 12%*v*/*v* DMSO (Sigma, Burlington, MA, USA)) and stored at −80 °C. In vitro differentiation was carried out as previously reported in RPMI-1640, 10%v/v FCS, 10 mM HEPES (Sigma), 1 mM sodium pyruvate (Thermo Fisher Scientific, Waltham, MA, USA), 1X MEM non-essential amino acids (Thermo Fisher Scientific), 10 U/mL penicillin, 10 mg/mL streptomycin, 2 mM L-glutamine (Sigma), and 50 ng/mL human macrophage colony-stimulating factor (M-CSF, Peprotech; London, UK). 

### 4.4. Foam Cell Formation

PBMC-derived macrophages were plated as 1 × 10^4^ cells/well in 200 μL medium in a flat-bottom 96-well plate and left to rest O/N. Thy next day, these were stimulated with 10 μg/mL oxidized LDL (oxLDL, Invitrogen, Thermo Fisher Scientific) for 24 h. After stimulation, all cells were stained with Oil Red O (Sigma-Aldrich, Burlington, MA, USA) as previously described [57]. The cells were imaged under an Axiovert 40 CFL inverted microscope (ZEISS, Oberkochen, Germany).

### 4.5. Statistical Analysis

Statistical analysis was performed using GraphPad Prism 9 (San Diego, CA, USA). Independent sample groups were first assessed for normality and equality of variances. As appropriate, for multiple treatment/group analyses, either parametric one-way analysis of variance (ANOVA, San Francisco, CA, USA) followed by Bonferroni post hoc selected comparisons of group means, or a non-parametric Kruskal–Wallis test and Dunn’s comparison post tests were performed, respectively. For analysis of two independent groups, Mann–Whitney non-parametric analysis was used in all cases. The relationship between sortilin levels, age-related metrics and the duration of disease was assessed via linear regression analysis performed in GraphPad Prism.

## 5. Conclusions

It is widely accepted that the peripheral immune system plays a significant role in the pathogenesis of Parkinson’s disease (PD). Our results reveal alterations in the immune cell populations of PD patients, including a significantly lower number of blood monocytes that are characterized by a higher activation state. Furthermore, blood monocytes and PBMC-derived macrophages from PD patients express high levels of sortilin, which correlates with high foam cell formation in vitro. These findings suggest that sortilin may be a potential biomarker of PD and a potential putative therapeutic target to inhibit atherosclerotic plaque formation observed in PD patients. Further research is warranted to elucidate the molecular mechanisms underlying the role of sortilin in innate immune cells in relation to PD and to explore the potential therapeutic implications of targeting specific immune cell populations. Multidisciplinary approaches combining immunology, neurobiology, and bioinformatics are likely to yield the most fruitful insights into the intricate relationship between the immune system and PD.

## Figures and Tables

**Table 1:** Participants for our studies were recruited from the outpatient clinic at General Hospital of Larisa, Thessaly, Greece.

Parkinson’s Disease (PD) Patients									
No	Sex	PD Type	Age	Age at Onset	Duration of Disease	BMI	UPDRS/Motor Scale	Drug Treatment	Comorbidities
1	F	*SNCA* (A53T)	55	40	15	16.9	29	Levodopa, Memantine Amantantin, antidepressants	PD dementia
2	M	Sporadic	42	31	11	23.5	25	Levodopa therapy, deep brain stimulation	None
3	M	Sporadic	60	59	1	25.3	8	Levodopa, Rasagelin	None
4	F	Sporadic	68	66	2	29	8	Dopamine agonist, Rasagelin	None
5	M	*SORT1* (G379W)	69	66	3	28.4	8	Dopamine agonist, Safinamide, Levodopa, ACE inhibitors	Hypertension
6	F	Sporadic	72	64	8	20.8	30	Levodopa, Memantine	PD dementia
7	M	Sporadic	68	64	4	26.6	9	Dopamine agonist, Rasagelin	Atrial fibrillation
8	F	*SNCA* (A30P)	67	62	5	28.1	18	Levodopa, angiotensin II receptor blockers	Thyroidectomy Hypertension
9	F	*SORT1* (G379W)	77	75	3	26	12	Dopamine agonist, Levodopa, Safinamide	None
10	M	*SORT1* (G379W)	60	57	3	30.4	12	Dopamine agonist, Levodopa	Stroke
**Healthy donors**									
No	Sex	Age		BMI		Drug treatment		Noted minor conditions	
1	M	22		22.7		no		no	
2	M	75		22.1		ACE inhibitors		Hypertension	
3	F	60		26.6		no		no	
4	M	36		27.8		no		no	
5	M	52		21.9		Atorvastatin, Rosurvastatin		Hyperlipidemia	
6	F	38		21.5		no		no	
7	M	68		27		no		no	
8	F	60		23.4		no		no	
9	F	67		25.4		no		no	
10	M	74		25.8		no		no	
11	M	75		24.8		no		no	

## Data Availability

Data generated or analyzed during this study are included in this published article and Supplementary Materials.

## Supplementary Materials

The following supporting information can be downloaded at: https://www.mdpi.com/article/10.3390/ijms25031791/s1.
