# Supplementary material for: Sortilin Expression Levels and Peripheral Immunity: A Potential Biomarker for Segregation between Parkinson’s Disease Patients and Healthy Controls
Source: Int J Mol Sci. 2024 Feb 1;25(3):1791. doi: 10.3390/ijms25031791 (PMC10855941; doi:10.3390/ijms25031791)
Supplement: Supplementary file 1 [file ijms-25-01791-s001.zip › ijms-2791123-supplementary.pdf]

Sortilin expression levels and peripheral immunity: A potential biomarker for segregation between Parkinson's Disease Patients and Healthy Controls

Maria Georgoula[1$,] Panagiotis Ntavaroukas[1$], Anastasia Androutsopoulou[1], Georgia Xiromerisiou[2,] Fani Kalala[3], Matthaios Speletas[3], Eftihia Asprodini[4], Anna Vasilaki [5], and Stamatia Papoutsopoulou[1,*]

Supplementary Table 1. The percentage of $CD3^+$ T cells, $CD19^+$ B cells and $CD14^+$ monocytes in freshly prepared peripheral blood detected by flow cytometry from healthy (H, n=11) and PD donors (PD, n=10).

Sample ID	$CD3^+$ T cells	$CD19^+$ B cells	$CD14^+$ Monocytes
H1	15.6	1.7	3.8
H2	14.2	1.3	3.2
H3	13.1	2.3	2.6
H4	14.8	4.6	1
H5	12.2	3	2.5
H6	14.8	2.2	2.5
H7	20.8	1.9	3.9
H8	13.4	1.5	3.6
H9	13.6	0.4	4.2
H10	11.1	1.1	2.8
H11	23.6	2.2	4.2
PD1	0.6	0	0
PD2	17.7	0	0.2
PD3	1.7	0	0.3
PD4	13.7	0	0
PD5	3.5	0	0.6
PD6	9.3	1.6	2.3
PD7	28	6.7	3.9
PD8	9.1	1.6	1.1
PD9	14.2	1.4	1.2
PD10	9.1	0.8	2.1

Analysis of the peripheral blood populations by flow cytometry

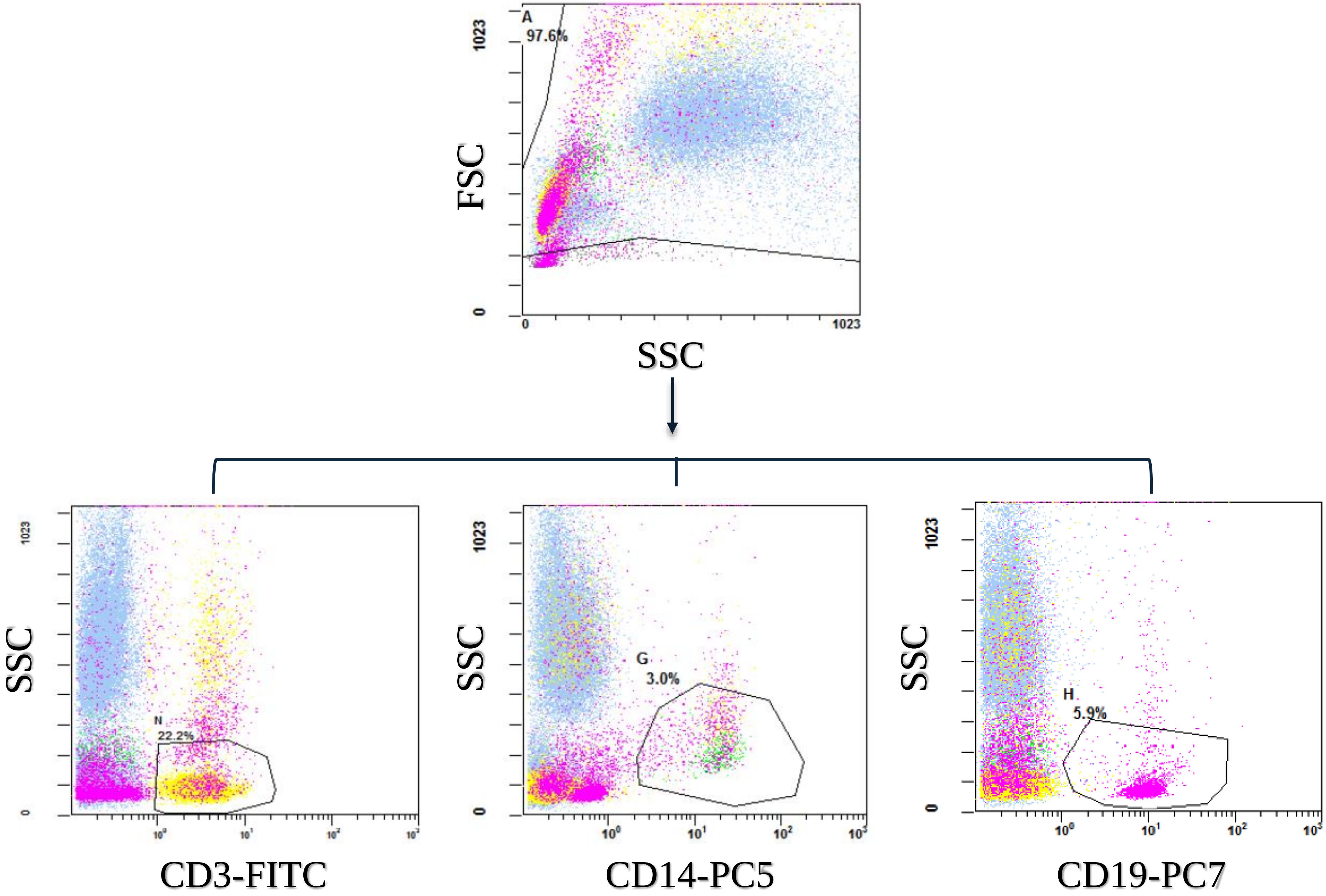

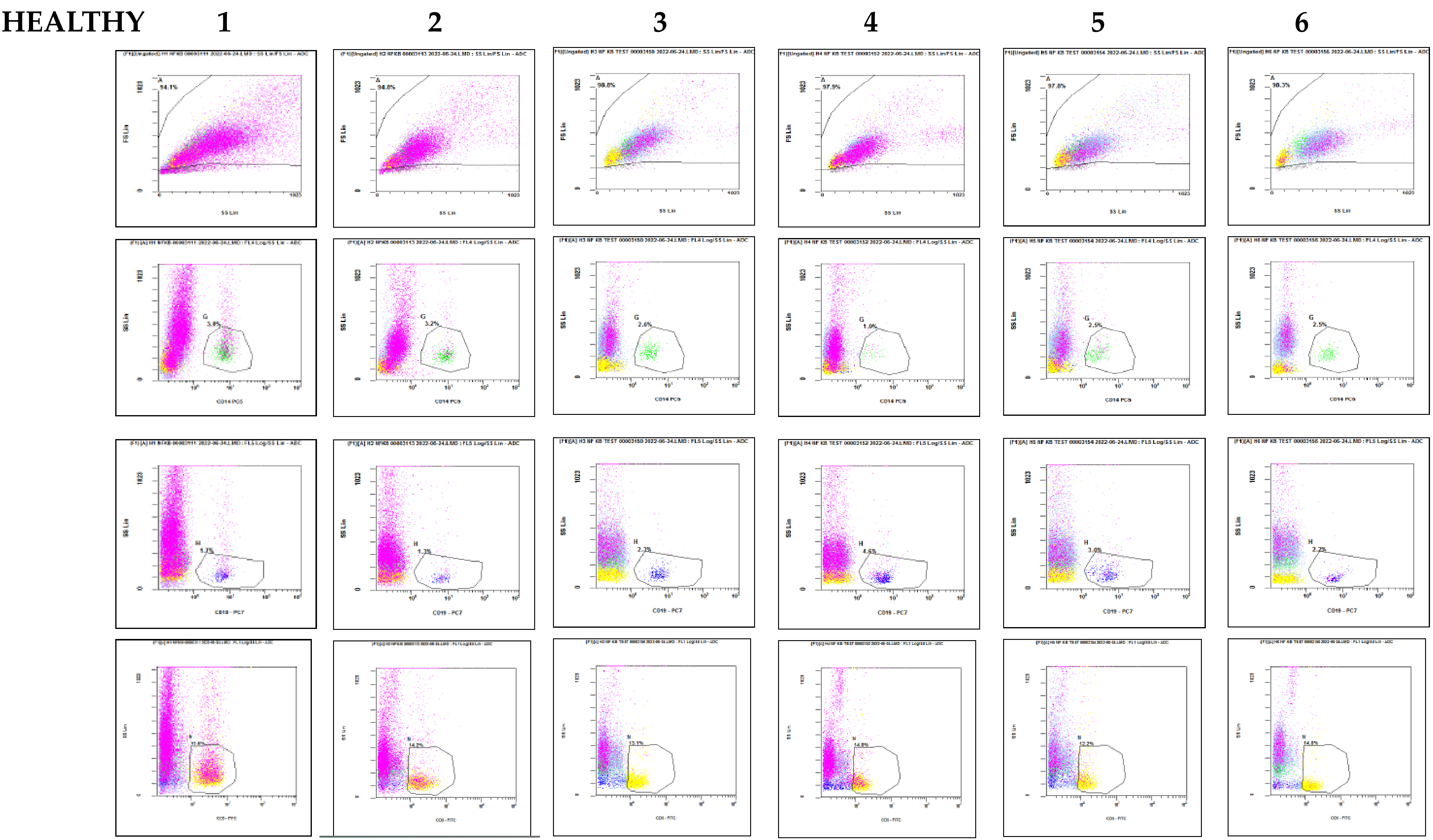
HEALTHY
1
2
3
4
5
6
FS Lin
SS Lin
CD14 PC5
CD19 - PC7

HEALTHY **7** **8** **9** **10** **11**

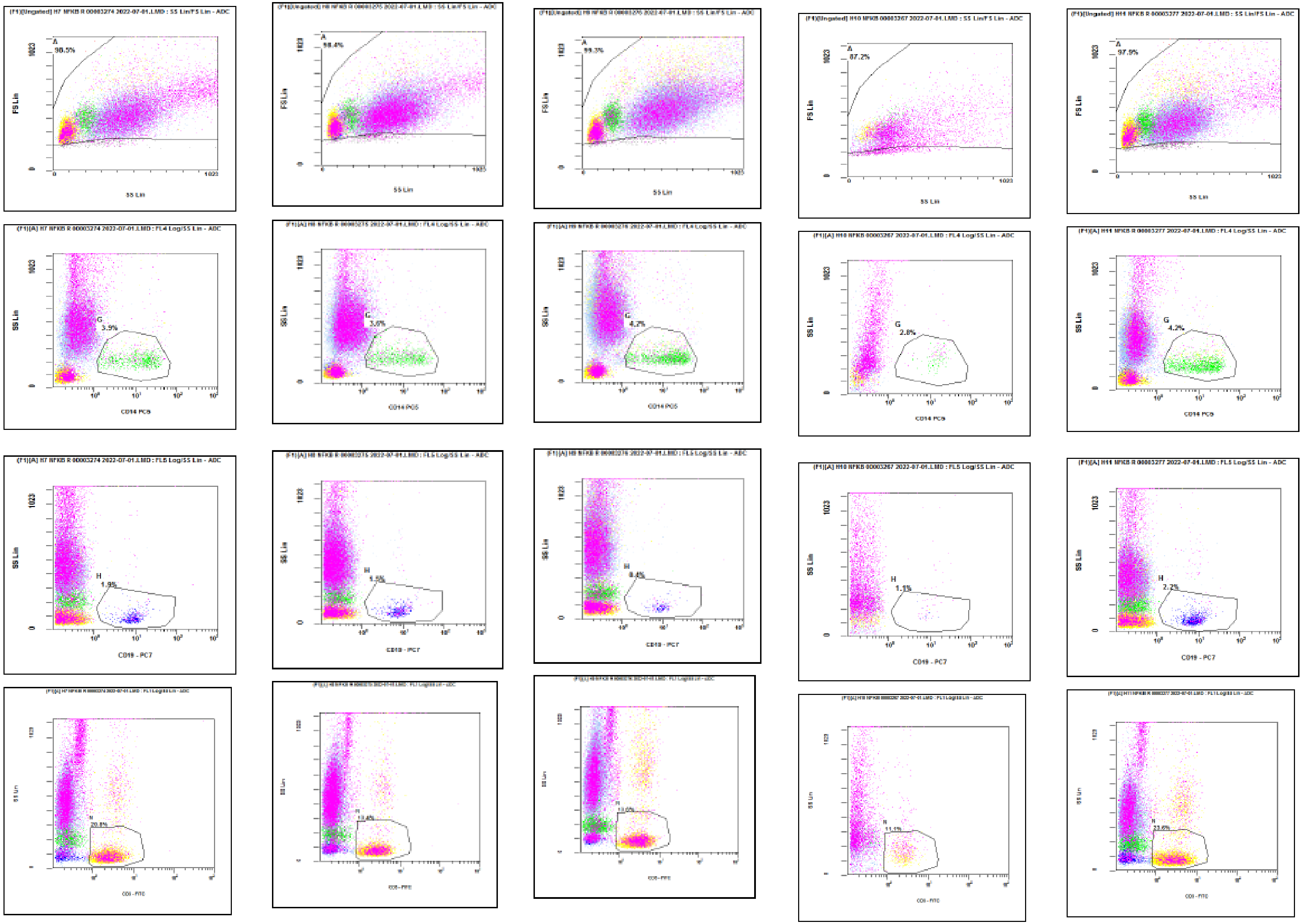

PD

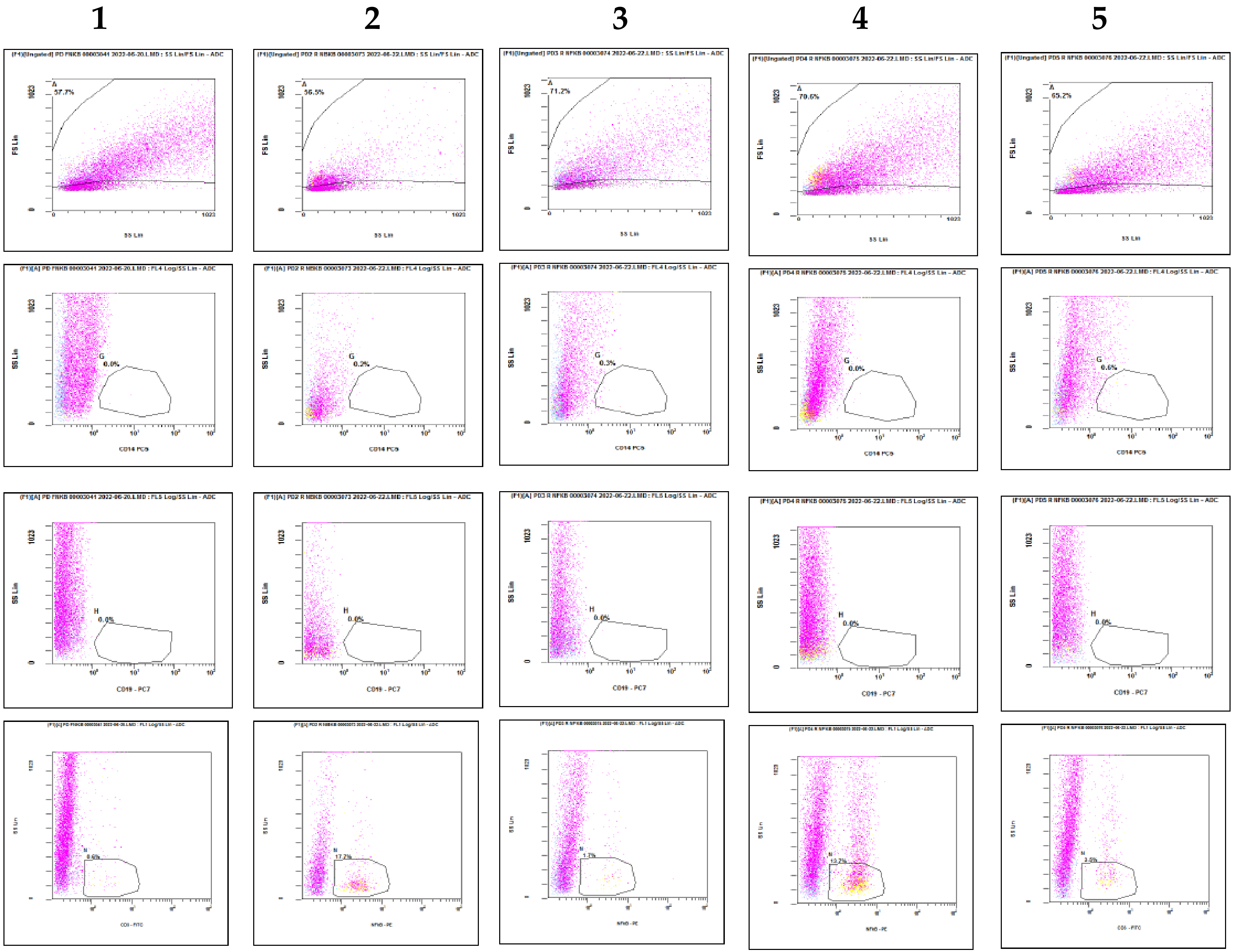

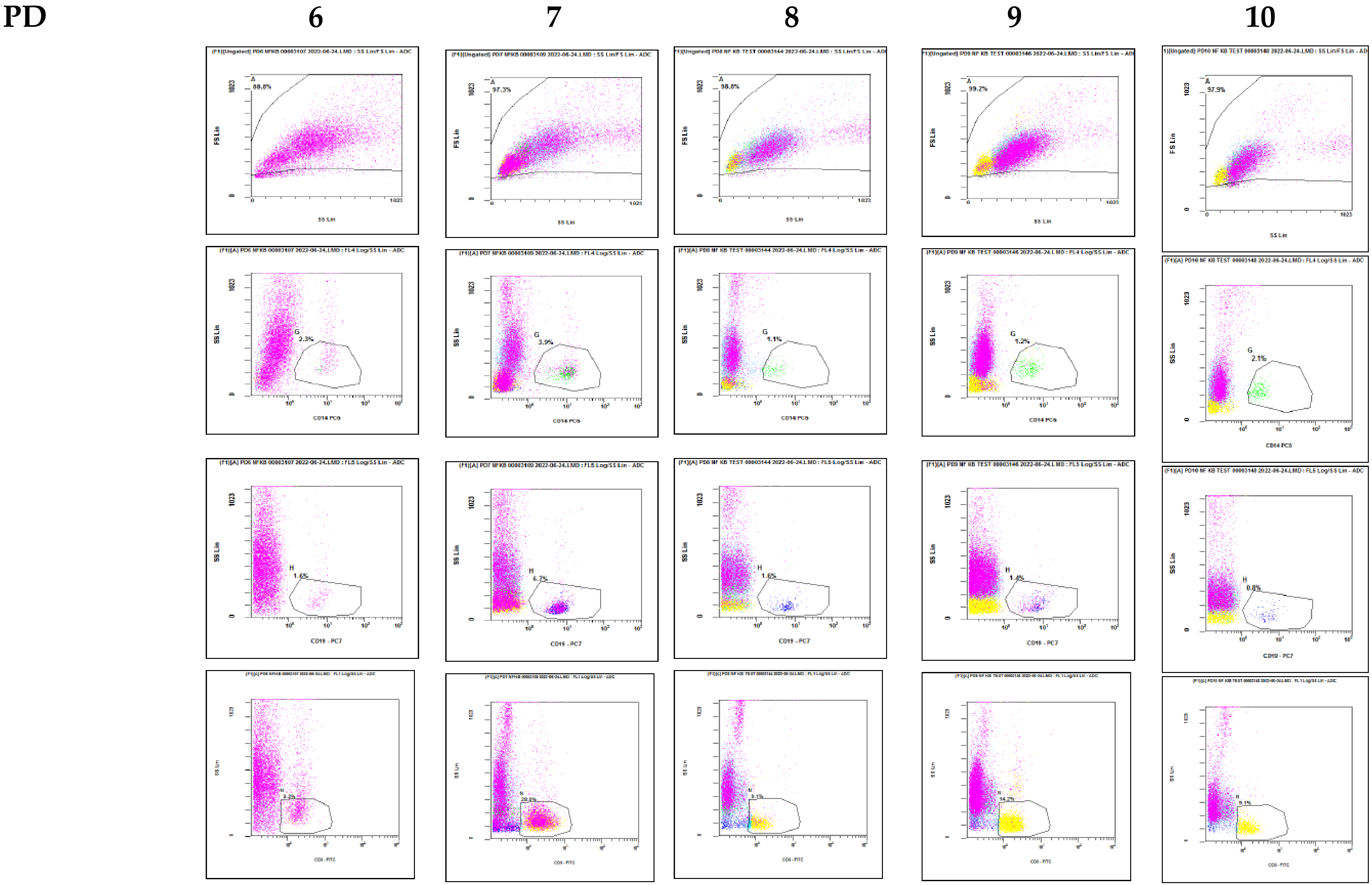
PD
6
7
8
9
10

Supplementary Figure 1. Gating strategy for the peripheral blood immune cell population analysis. The CD3+, CD19+ and CD14+ populations are expressed as percentage of total peripheral blood cells. Data for all donors are presented

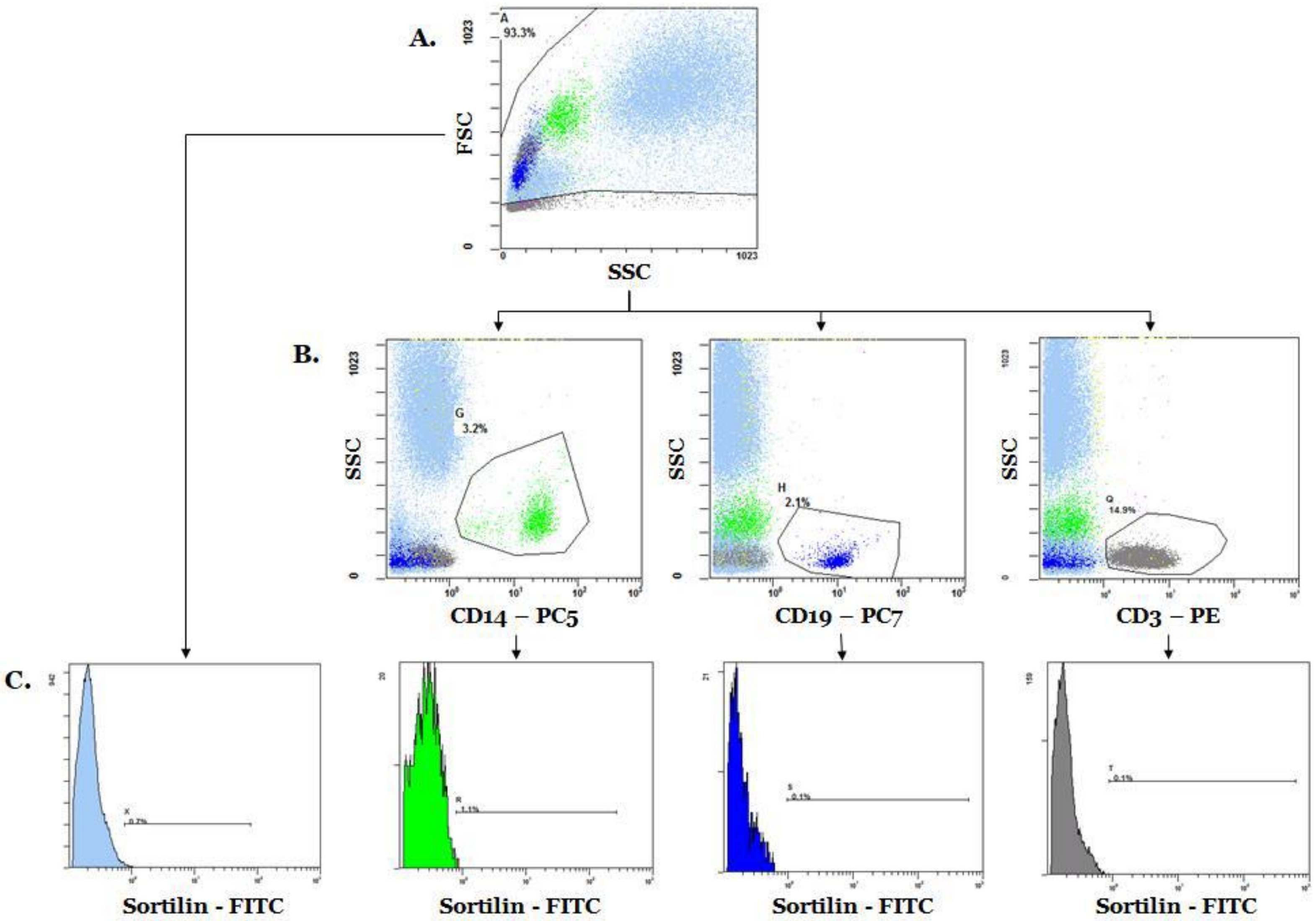

Supplementary Figure 2. Gating strategy for sortilin expression on the peripheral blood immune cell populations, specifically CD3+ T cells, CD19+ B cells and CD14+ Monocytes.

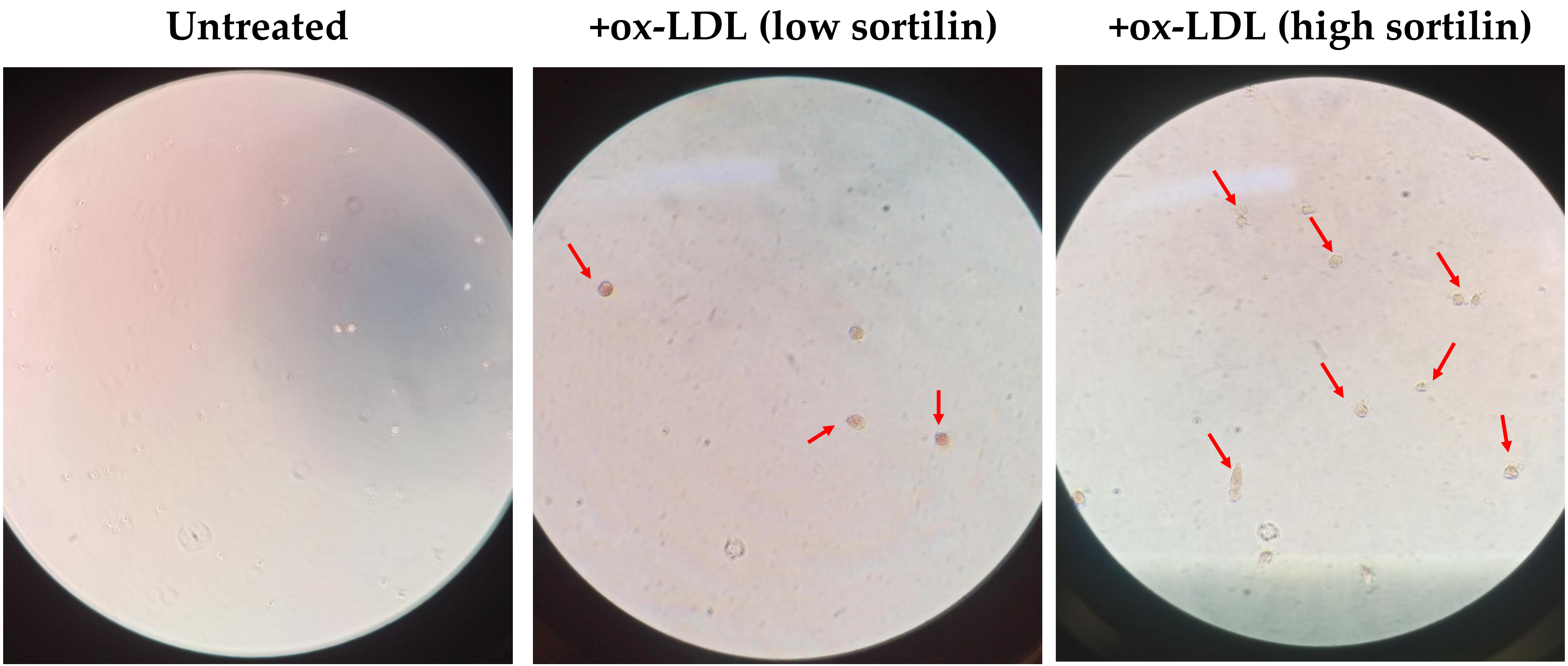

Supplementary Figure 3. Oxidized LDL-induced foam cell formation in human PBMC-derived macrophage cultures. Macrophage cultures were either left untreated or stimulated with 10μg/ml ox-LDL for 24h. Cell were stained with Oil Red O and the number of foam cells was calculated based on at least 50 cells, obtained by randomly selected fields. Images from low sortilin-expressing cells and high sortilin-expressing cells are shown. Red arrows point at foam cells.
